# Improving mental health among intensive care unit staff with communication skills training

**DOI:** 10.3389/fpsyg.2024.1454702

**Published:** 2024-10-14

**Authors:** Johan Holmberg, Ingvar Rosendahl, Rebecca Andersson, Mike K. Kemani, Linda Holmström, Lars-Göran Öst, Rikard K. Wicksell

**Affiliations:** ^1^Centre for Psychiatry Research, Department of Clinical Neuroscience, Karolinska Institutet and Stockholm Health Care Services, Stockholm, Sweden; ^2^Department of Clinical Neuroscience, Karolinska Institutet, Stockholm, Sweden; ^3^Medical Unit Medical Psychology, Women's Health and Allied Health Professionals Theme, Karolinska University Hospital, Stockholm, Sweden; ^4^Department of Psychology, Stockholm University, Stockholm, Sweden; ^5^Pain Clinic, Capio St Göran Hospital, Stockholm, Sweden

**Keywords:** intensive care units, healthcare professionals, communication skills training, mental health, occupational stress, work engagement, psychological flexibility

## Abstract

**Purpose:**

Intensive Care Unit (ICU) staff report that a large part of the work-related distress they experience is related to communication situations with colleagues, patients, and relatives. Based on these findings, the aim of the present study was to preliminary evaluate the effects of a novel Communication Skills Training (CST) program on mental health among ICU staff.

**Methods:**

The CST program was delivered to the entire work force of an ICU at a Swedish hospital and was evaluated as an uncontrolled clinical trial with three repeated measures. 100 participants were eligible and included in the analyses. The theoretical framework for the program was learning theory and cognitive behavioral therapy. The program was delivered by two psychologists and included one full-day lecture and three subsequent two-hour supervision sessions. Changes in mental health was evaluated with self-report questionnaires measuring perceived stress, general mental health, work engagement, and psychological flexibility.

**Results:**

Analyses of three repeated measures showed significant improvements with 1.3 points lower perceived stress and 2.0 points lower general mental health after the intervention. No differences were seen in measures of work engagement or psychological flexibility.

**Conclusion:**

Findings of the study supports the utility of the CST program as an intervention to target perceived stress and general mental health within intensive care. These findings should be further validated in trials with improved design.

## 1 Introduction

High work-related demands in Intensive Care Units (ICUs) have been observed and reported in research literature since the 1960s. Although this issue has recently gained more attention due to the Corona virus disease-19 pandemic (COVID-19), concerns have been raised repeatedly even before the pandemic and severe health and performance-related consequences of work-related stress highlight the importance of research on preventive strategies targeting the specific challenges in intensive care.

Intensive care medical staff report high levels of burnout, traumatic stress, symptoms of anxiety, depression and fatigue (Mealer et al., 2009; Mealer et al., 2012; van Mol et al., 2015). In addition to health problems, there are work-related consequences, e.g., turnover rates (Adriaenssens et al., 2015), intention to leave (Khan et al., 2019; Stone et al., 2006), and reduced work performance (van Mol et al., 2015). The specific stressors shown to be associated with health-related consequences include conflicts with co-workers (Azoulay et al., 2009; Crickmore, 1987; Elshaer et al., 2018; Embriaco et al., 2007; Martins Pereira et al., 2016), conflicts with patients and/or their families (Crickmore, 1987; Martins Pereira et al., 2016), workload (Crickmore, 1987; Embriaco et al., 2007; Vandevala et al., 2017), moral distress (Martins Pereira et al., 2016; Piers et al., 2011), and issues of life and death (Martins Pereira et al., 2016). In a multicenter study, Martins Pereira et al. (2016) found that conflicts were the most significant determinant of burnout syndrome and that having a post graduate education in intensive care was a protective factor. Furthermore, an extensive evaluation of the prevalence of conflicts in intensive care was done by Azoulay et al. (2009) who obtained data of perceived conflicts from 7,498 staff members from 323 ICUs in 24 countries. The study showed that 70% of ICU staff experienced conflicts in the past week, 50% of the conflicts were perceived as serious or dangerous, and conflicts within the team accounted for the majority of conflicts. Also, poor communication in general or during end-of-life care, was perceived as a common source of conflict. The authors concluded that workload, communication and end-of life care could be potential targets for interventions.

Thus, demands in intensive care and their implications call for interventions promoting health and work performance. This was even more imminent during COVID-19 when ICU staff were exposed to increased demands (Appelbom et al., 2021; Monti et al., 2023; Rossettini et al., 2021). However, studies evaluating interventions in intensive care settings are few, and knowledge on the effects of interventions are needed. A review including 12 studies published between 2011 and 2019 that addressed stress management interventions among intensive and critical care nurses was done by Alkhawaldeh et al. (2020). The authors concluded that interventions based on cognitive behavioral therapy and mindfulness can reduce stress reactions. Notably, only two studies utilized a randomized controlled design, limiting the validity of the results. Furthermore, in a systematic review of the prevalence of emotional distress in intensive care van Mol et al. (2015) suggest communication skills, ethical rounds, and mindfulness as possible starting points for the development of prevention strategies. Referring to three studies focused on communication the authors report significant reductions in burnout and depression (Quenot et al., 2012), improved communication, and significantly reduced emotional exhaustion (Sluiter et al., 2005) and reduced physical and mental effort (Loiselle et al., 2012). Again, non-randomized and non-controlled designs were used so the results are considered preliminary.

In sum, previous studies illustrate that team communication is an important source of distress suggesting the utility of interventions targeting communication. Given the current demand on frontline staff combined with a lack of studies evaluating the benefit of preventive interventions, more research is urgently needed. Therefore, a Communication Skills Training (CST) program was developed that included the opportunity to identify and discuss common communication problems and practice key communication skills by applying principles from learning theory and cognitive behavioral therapy. The aim of this present study was to preliminarily evaluate the effects of this CST program on mental health among intensive care medical staff. More specifically, the study hypotheses were that the CST program would show improvement on measures of occupational stress, general mental health, work engagement, and psychological flexibility.

## 2 Materials and methods

### 2.1 Participants

Following a request from the managers of an ICU in a Swedish hospital, the CST program was developed and provided to the staff as part of an initiative to manage occupational stress. Inclusion was based on employment and all staff at the unit were considered eligible to participate in the research study. This included nurses, physicians, assistant nurses, management, and administrative staff. There were no exclusion criteria applied. Recruitment was done at the beginning of the program at the hospital where the ICU was located. The study was approved by the Swedish Ethical Review Authority (Registration number 2014/42-31/3 and 2015/1881-32/3), all methods were performed in accordance with relevant guidelines and regulations, and informed consent was obtained from all participants. Participation in the study was not required to participate in the CST and participants were informed that they could withdraw their consent at any time.

### 2.2 Design

**Figure 1:**
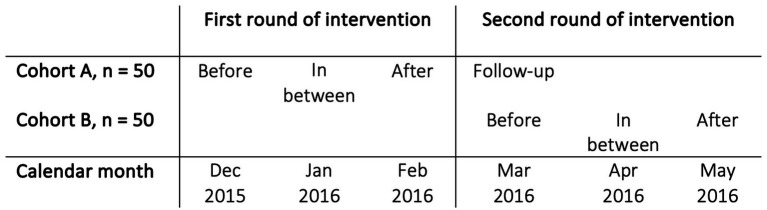
Description of each measurement occasion of the intervention to the two cohorts, A and B.

The evaluation of the CST program was conducted as an uncontrolled clinical trial with three repeated assessment occasions. The workforce was divided into two cohorts, that received the intervention sequentially, see Figure 1. The cohorts were then aggregated and evaluated as one group. Data consisted of self-report questionnaires administered at before the intervention, in between, and after the intervention. Two psychologists (co-author RG and another clinical psychologist) delivered the intervention and collected the data. Due to logistical issues and clinical demands, follow-up assessments were completed only by cohort A.

### 2.3 Content and delivery of intervention

The CST program (see Supplementary material, Content of training and supervision) was delivered in 2015/2016 by two psychologists with training in Cognitive Behavioral Therapy (CBT; O’Donohue and Fisher, 2009; Richardson and Rothstein, 2008), Acceptance and Commitment Therapy (ACT; Hayes et al., 2012), and Motivational Interviewing (MI; Miller and Rollnick, 2012). It consisted of one full-day lecture (6 h) in a large group format, followed by three 2-h small group session (approx. 10 participants) scheduled to enable participants to work on assignments focused on work-related communication between sessions. The entire program was delivered within 3 months in locations at the hospital close to the ICU so that participants could easily attend the CST program.

The full-day lecture covered basic aspects of behavior change and key communication skills and also provided opportunities to apply these skills. The training was based on learning theory, using interventions from CBT, ACT and MI. To achieve learning of specific skills, role play was used to facilitate experiential learning that included emotional, cognitive as well as behavioral features.

During the subsequent supervision, participants were encouraged to discuss communication problems that arise in their work roles as well as strategies to address them. More specifically the training consisted of: (1) value clarification in relation to patients and colleagues; (2) analysis of the consequences of avoidance behavior in relation to work-related discomfort; and (3) training in communication skills.

### 2.4 Assessments

Four self-report questionnaires were used to assess different aspects of mental health among the ICU staff. The Perceived Stress Scale-10 (PSS-10) to assess occupational stress, the General Health Questionnaire-12 (GHQ-12) to assess general mental health primarily depression, The Utrecht Work Engagement Scale (UWES) to assess work engagement, and finally the Work-related Acceptance and Action Questionnnaire (WAAQ) to assess psychological flexibility.

The Perceived Stress Scale (Cohen et al., 1983) was developed as a measure of the subjective psychological aspect of stress. An example of an item is “In the last month, how often have you felt that you could not cope with all the things you had to do?.” Items are rated on a five-point Likert scale from *never* (0) to *very often* (4). The scoring gives a total sum between 0 and 40. A Swedish validation of PSS-10 (Nordin and Nordin, 2013) showed adequate psychometric properties. In the current sample, Cronbach’s alpha was 0.85.

The General Health Questionnaire (Goldberg and Hillier, 1979) measures general mental health and psychological distress. Although it has been used primarily as an instrument to detect depression in primary care, it has also been used to measure change during treatment. An example of an item is “I have recently lost much sleep over worry.” On a four-point Likert scale items are rated from 0 to 3, resulting in a total score of 0 to 36. This scoring method has been recommended for use with group comparisons and parametric analyses (Banks et al., 1980). A Swedish validation of GHQ-12 (Lundin et al., 2016) showed good internal consistency with Cronbach’s alpha of 0.83–0.89 (depending on scoring method). In the current sample, Cronbach’s alpha was 0.79.

The Utrecht Work Engagement Scale (Schaufeli and Bakker, 2004) was developed to measure work engagement, defined as “positive, fulfilling, work-related state characterized by vigor, dedication, and absorption.” (Schaufeli et al., 2002). An example of an item is “I’m enthusiastic about my job.” Seventeen items are rated on a seven-point Likert scale from *never* (0) to *always/every day* (6), and the sum divided by number of items gives a score between 0 and 6. A Swedish validation of UWES (Hallberg and Schaufeli, 2006) showed good internal consistency with a Cronbach’s alpha of 0.93. In the current sample, internal consistency according to Cronbach’s alpha was 0.90.

The Work-related Acceptance and Action Questionnaire (WAAQ; Bond et al., 2013) was developed to measure psychological flexibility, defined as the ability to act in accordance with chosen goal even in the presence of disturbing thoughts and feelings (Hayes et al., 2012). An example of an items is “I can work effectively, even when I doubt myself.” Seven items are rated on a seven-point Likert scale from *never true* (1) to *always true* (7) and the total score range from 7 to 49. A Swedish validation of the WAAQ among healthcare professionals (Holmberg et al., 2019) showed good internal consistency (Cronbach’s alpha 0.85) and good test–retest reliability (ICC 0.85). In the current sample, Cronbach’s alpha was 0.87.

### 2.5 Data analysis

Statistical analyses were conducted using the Statistical Packages for Social Sciences (SPSS), version 26 (IBM Corp, 2019) and R (R Core Team, 2021). The specific packages used in R were nlme (Pinheiro et al., 2013) to conduct Linear Mixed Models (LMM), and ggplot2 to create figures (Wickham, 2009).

Missing data were analyzed by summarizing for each participant the number of missing values for the four outcome variables. The number of missing values was then dichotomized to any number of *missing* vs. *no missing* and used as a dependent variable in a logistic regression to investigate if any other variable given at the start of the study predicted the risk of missing data.

Descriptive statistics of participants’ background characteristics were tabulated as means and standard deviations or as frequencies and percentages. Background variables included pre-intervention levels of outcome variables along with information on age, years of work experience, sex, and profession.

Difference between pre- and post-measure and effect of time. Means and standard deviations for the four outcome variables in the study before, in between and after the intervention were tabulated with dependent *t*-statistics and Cohen’s *d* of differences from before to after. Dependent pairwise t-test analyses were performed with listwise deletion of missing cases. The effects of intervention were further evaluated by calculating fixed effect estimates of measurement occasion in linear mixed models (LMM) with three repeated assessments. Measurement occasion was treated as a categorical variable in the models, resulting in distinct estimates of the time effects for each of the two follow-up measurements instead of linear average trends. LMM analysis was chosen to maximize the size of sample by including all possible data.

## 3 Results

### 3.1 Participants

Demographic characteristics and initial levels of mental health for the participants are shown in Table 1. Although differences in initial levels of mental health between professional groups were analyzed, the number of participants in the subgroups was considered too low for valid results. Notably, pre-treatment levels of occupational stress, general mental health, and engagement were within the normal range, as measured by normative data from the PSS-10 (Nordin and Nordin, 2013), the GHQ-12 (Cohen and Williamson, 1988) and the UWES (Hallberg and Schaufeli, 2006).

**Table 1:** Demographic characteristics and initial levels of occupational psychological health of the study participants (*n* = 100).

Continous characteristics	Mean	Standard deviation	Missing information
Age	47.5	9.2	8
Years of work experience	19.7	11.1	11
PSS-10	13.4	5.9	5
GHQ-12	8.2	5.1	5
WAAQ	36.5	5.4	5
UWES	4.1	0.8	5
Categorical characteristics	Number	Proportion	
Sex			
Females	79	85.9	8
Males	13	14.1	
Profession			
Nurse	53	53.5	1
Assistant nurse	33	33.3	
Physician	7	7.1	
Miscellaneous	6	6.1	

PSS-10, Perceived Stress Scale-10; GHQ-12, General Health Questionnaire-12; WAAQ, Work-related Acceptance and Action Questionnnaire; UWES, Utrecht Work Engagement Scale.

Pearson bivariate correlation coefficients were calculated to evaluate the relationship between mental health variables and background variables. Results showed significant negative correlations between age and PSS-10 (*r* = −0.30, *p* = 0.005), and years of work experience and PSS-10 (*r* = −0.32, *p* = 0.003). Also, a significant positive correlation was seen between years of work experience and WAAQ (*r* = 0.22, *p* = 0.041). The remaining coefficients were small and non-significant.

### 3.2 Missing data

In total, 105 out of 124 individuals signed informed consent and were enrolled in the study. Five participants provided no information on any of the four outcome measures and were therefore considered as withdrawn participants and excluded from the study. Thus, analyses were based on 100 participants.

Of the 100 study participants, 71 provided a complete set of data, i.e., all four outcome measures at all time points. One baseline characteristic (dummy coded, 1 = being a physician or 0 = not being a physician) showed a significant increased risk for having missing information on any of the four outcomes with an odds ratio of 10.8, *p* = 0.009.

### 3.3 Evaluation of changes over time in levels of mental health

Levels of mental health at each time point are shown in Table 2, including *t*- and *p*-values of differences between before- and after-measurements, as well as Cohen’s *d* and 95% confidence intervals of Cohen’s *d*.

**Table 2:** Group means of occupational stress, general mental health, engagement, and work-related psychological flexibility at before-, in between-, and after-intervention, along with before- to after-measurement differences.

Outcome	Before	In between	After	Difference before-after^a^	*t*-value (degrees of freedom)	*p*-value	Cohen’s *d* (95% Confidence interval)
PSS-10							
Mean	13.4	13.0	12.3	1.3	2.61 (77)	0.011*	0.225 (0.053; 0.398)
Standard deviation	5.9	5.8	5.7	4.4			
Denominator	95	89	82	78			
GHQ-12							
Mean	8.2	8.0	6.5	2.0	4.62 (76)	<0.001***	0.404 (0.224; 0.584)
Standard deviation	5.1	4.9	4.2	3.8			
Denominator	95	89	81	77			
WAAQ							
Mean	36.5	36.3	36.5	0.3	0.66 (77)	0.511	0.056 (−0.111; 0.223)
Standard deviation	5.4	5.9	5.3	4.0			
Denominator	95	89	82	78			
UWES							
Mean	4.1	4.1	4.0	0.1	0.76 (74)	0.452	0.062 (−0.100; 0.223)
Standard deviation	0.8	0.8	0.7	0.5			
Denominator	95	89	79	75			

*p < 0.05, **p < 0.01, *** < 0.001. PSS-10, Perceived Stress Scale-10; GHQ-12, General Health Questionnaire-12; WAAQ, Work-related Acceptance and Action Questionnnaire; UWES, Utrecht Work Engagement Scale.

a Observe that values refer to within-group differences based on participants providing assessments both at before- and after-measurement, see associated denominators which consequently differ from previous columns.

The dependent t-test statistic showed significant differences in PSS-10 (Mean of differences = 1.31, 95% CI = 0.31–2.31) and GHQ-12 (Mean of differences = 2.01, 95% CI = 1.14–2.86). The size of effects (Cohen’s *d*) were 0.225 (95% CI = 0.053; 0.398) for PSS-10 and 0.404 (95% CI = 0.224; 0.584) for GHQ-12, respectively. Differences in self-reported WAAQ and UWES were small and non-significant.

**Figure 2:**
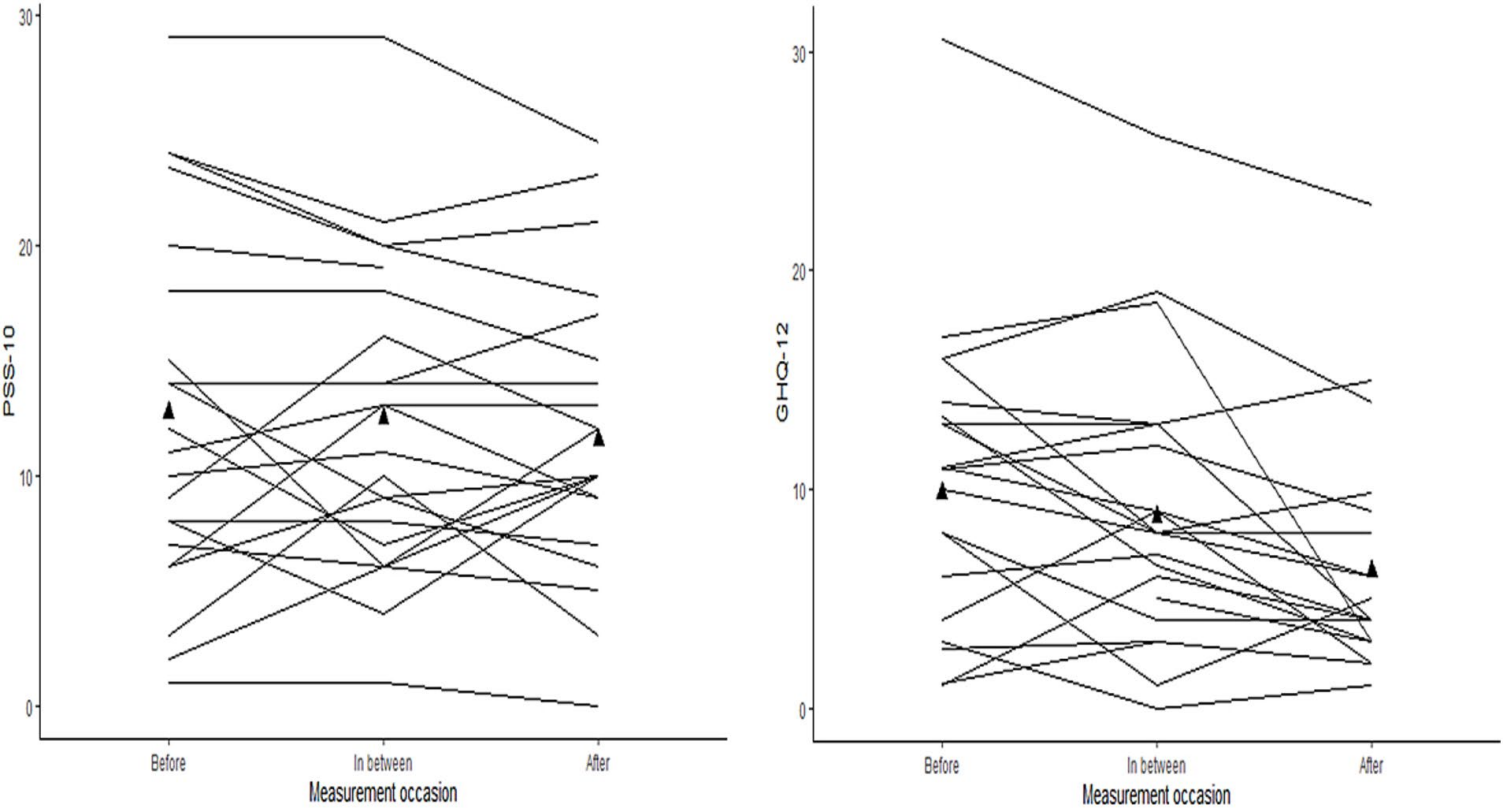
Trajectories of PSS-10 and GHQ-12 for a random sample (*n* = 25) of the participants. Group mean level of PSS-10 and GHQ-12 are marked with ▲ at the measurement occasions before, in between, and after.

Next, linear mixed models (LMM) analysis were performed for the two dependent variables PSS-10 and GHQ-12 to evaluate the changes in scores at each follow-up measurement compared to before scores with fixed effect of time and random effects of individual participants in models with three repeated measures. The random intercept part of the models allowed for an expected variation in baseline levels of mental health. First, individual trajectories were plotted in line graphs for a random sample (25%) of the participants to get an overview of individual variation in before values and development over time, for PSS-10 and GHQ-12 (Figure 2).

Figure 2 displays similar patterns for PSS-10 and GHQ-12 with different initial values and different developments between participants during follow-up. Moreover, the intraclass correlation (ICC) values from an empty LMM model, i.e., a model without any predictors, were 0.75 for PSS-10 and 0.66 for GHQ-12, with both values indicating the necessity of taking the variance of between-participants’ initial values into account in the following analyses. Next, comparisons between models with increasing complexity were made by analysis of variance (ANOVA). For both outcomes (PSS-10 and GHQ-12), the fit indices and the likelihood-ratio tests showed that random intercept models with time as fixed effect predictors were significant improvements compared to basic models with fixed effects only, *χ*^2^ (1) = 7.16, *p* = 0.028 for PSS-10 and *χ*^2^ (1) = 21.86, *p* < 0.001 for GHQ-12. Therefore, the analysis of an effect of time for PSS-10 and GHQ-12 was accordingly estimated with random intercepts and time treated as categorical fixed effects. The results from the final LMM models for PSS-10 and GHQ-12 are displayed in Table 3.

**Table 3:** Results from two linear mixed model analyses, i.e., Perceived Stress Scale-10 (PSS-10) and General Health Questionnaire-12 (GHQ-12), with measurement occasion and years of work experience as independent variables in the model.

Scale	Estimate	Standard error	Degrees of freedom	*t* value	*p* value	95% Confidence interval
PSS-10						
Fixed effects						
Intercept	13.43	0.59	143	22.76	<0.001	12.27; 14.58
In between measurement	−0.50	0.45	143	−1.11	0.268	−1.39; 0.39
After measurement	−1.22	0.47	143	−2.59	0.011	−2.14; −0.30
Years of work experience	−0.13	0.04	87	−2.74	0.008	−0.23; −0.04
Random effects						
Standard deviation (Intercept)	4.69					3.96; 5.55
GHQ-12						
Fixed effects						
Intercept	8.07	0.49	142	16.37	<0.001	7.10; 9.03
In between measurement	−0.39	0.40	142	−0.99	0.324	−1.17; 0.38
After measurement	−1.52	0.41	142	−3.68	<0.001	−2.33; −0.71
Years of work experience	−0.08	0.04	87	−2.10	0.038	−0.16; −0.01
Random effects						
Standard deviation (Intercept)	3.85					3.25; 4.57

Results showed a significant fixed effect of time with decreased levels of −1.22 in PSS-10, and −1.52 in GHQ-12 at after measurement while the effect was smaller and non-significant at in between measurement for both scales (see Table 3).

Additionally, demographic variables were entered as covariates in the model to assess possible influence. As seen in Table 3, only years of work experience (centered around its group mean) had a significant influence on the effect of time on the changes in PSS-10 (point estimate −0.13) and in GHQ-12 (point estimate −0.08). Since work experience was centered around its mean, this result represents a positive change (decrease) in PSS-10 and GHQ-12 for participants with a work experience above the mean, and accordingly a negative change (increase in PSS-10 and GHQ-12) for participants with year of work experience below the group mean.

Table 3 shows that the confidence intervals for the standard deviation of the random intercept do not include zero for either PSS-10 or GHQ-12, suggesting that both scales varied significantly at baseline across the participants.

## 4 Discussion

The aim of this study was to initially evaluate the effects of a CST-program on changes in mental health among intensive care medical staff, using an uncontrolled clinical trial design with three repeated measures. Main results of the study from dependent *t*-tests and linear mixed model analyses showed significant improvements in measures of occupational stress and general mental health, i.e., Perceived Stress Scale-10 and General Health Questionnaire-12. Furthermore, linear mixed model analyses showed improved fit with random intercept models for both PSS-10 and GHQ-12, indicating the relevance of considering individual variation. Also, planned post-hoc analyses of the influence of demographic variables showed that years of work experience had a significant effect on changes in levels of occupational stress and general mental health. This means that the CST may have a positive impact on reducing occupational stress and improving general mental health. Additionally, participants with more years of work experience may find it easier to benefit from the intervention. Finally, results show no significant changes in psychological flexibility (Work-related Acceptance and Action Questionnaire) or work engagement (Utrecht Work Engagement Scale).

The results from this study support findings from previous research suggesting the utility of a CST-program in improving mental health in ICUs (van Mol et al., 2015). Previous studies focused on communications skills of ICU staff have shown positive effects on increased well-being and performance (Loiselle et al., 2012), reduction in burnout and depression (Quenot et al., 2012), and decreased emotional exhaustion (Sluiter et al., 2005). This study adds to previous knowledge by suggesting possible effects on reducing occupational stress and improving general mental health.

Notably, previous studies have lacked a control condition, providing only preliminary evaluations of the interventions. Similarly, the present study was conducted without a control condition, limiting our ability to draw conclusions about the causal effects of the intervention. Without an active control group, it is not possible to determine whether the observed effects can be directly attributed to the intervention. Factors such as the impact of being observed, seasonal variation in workload, or organizational changes—variables that could not be readily controlled for—cannot be ruled out. In addition, incorporating a reliable and valid measure of communication skills would help to better establish processes of change related to the intervention. However, recruiting all employees from a specific unit, rather than a selected or self-selected subset of the work force enhances the ecological validity and generalizability of the findings. Additionally, the use of repeated measures with three assessment points, along with linear mixed model (LMM) analysis, allowed for the inclusion of all participants who had at least one assessment point, thereby improving the representativeness of the sample.

Implications of this study are several. The variables of PSS-10 and GHQ-12 seems like useful instruments to evaluate differences and change of occupational stress and general mental health in a future randomized controlled trial. The effects of the intervention on PSS-10 and GHQ-12 were not observed at the first follow up measurement but at the second. This could mean that the length of the intervention, i.e., number of supervision sessions, might be an important variable and should be taken into consideration when designing similar interventions. In addition, we found that years of work experience had an influence on the observed effect. We do not know why this is, but it should be addressed in future design of interventions so that the intervention are fitted to be helpful to the complete workforce. At last, the preliminary positive results in general encourage additional studies focusing on communication skills training.

In conclusion, the present study adds to the growing body of studies evaluating interventions aimed at improving mental health among intensive care staff. Although tentative, the results support the utility of communication skills training for mental health in ICU staff, which should be further evaluated in larger studies with improved designs.

## Data Availability

The raw data supporting the conclusions of this article will be made available by the authors, without undue reservation.
