# Supplementary material for: Improving mental health among intensive care unit staff with communication skills training
Source: Front Psychol. 2024 Oct 14;15:1454702. doi: 10.3389/fpsyg.2024.1454702 (PMC11513591; doi:10.3389/fpsyg.2024.1454702)
Supplement: Supplementary file 1 [file Table_1.DOCX]

Supplementary Material: Content of Training and Supervision

| **Training** | **Content** |
| --- | --- |
| Introductory full-day lecture | - Overview of communication skills training in healthcare. - Use of applied behavioural analysis as a tool for improving communication skills. - Introduction, modelling and practice of five core communication skills.   - Active listening/validation   - Expressing appreciation   - Asking for help   - Assertiveness training, including how to say no   - Sharing mistakes at work - Introduction to experiential avoidance and its relevance in communication. Strategies for managing personal discomfort using acceptance and defusion techniques. - Clarification of personal and professional values in relation to patients and colleagues. - Analysis of the consequences of avoidance behaviors in handling work-related discomfort. - Assignment: Self-observation of personal communication behaviours, identifying areas for improvement to be addressed during supervision sessions. |
| **Supervision sessions** | **Content** |
| Session #1 | - Introduction to supervision, group rules, and agenda setting. - Review of the home assignment. - Analysis of challenging communication situations using learning theory. Identification of desirable behaviours based on long-term positive outcomes or alignment with personal values at work. - Role-playing of selected communication situations to practice desired behaviours. Peer feedback. - Assignment: (1) Formulate personal learning goals for supervision participation; (2) Practice each of the five core communication skills at least once before the next supervision. |
| Session #2 | - Follow up on individual assignments, with the supervisor reinforcing the application of communication skills learned in the programme. - Review and practice active listening skills as applied to everyday work situations. - Identification of personal values in the workplace. - Functional analysis of personal avoidance behaviours in an interactions with colleagues, patients, or their relatives (e.g., reluctance to ask for help with a medical task due to fear of embarrassment or insecurity). - Role-play of desirable communication behaviours previously analyzed. Practice acceptance to address personal discomfort that hinder value-driven behaviours. Peer feedback. - Assignment: Practice one or more of the five core communication skills multiple times per week, gradually in more challenging situations. |
| Session #3 | - Follow up on individual assignment, with reinforcement of communication skills from the programme. - Functional analysis of a communication scenario involving a colleague, patient or relative. Identification of appropriate communication skills aligned with values. - Role-play practice with peer feedback. - Summary of the content and objectives of this communication skills programme by the supervisor. - Introduction to ACT techniques (defusion) for managing stressful thoughts and feelings in communication. - Participants are invited to provide feedback on their experience in the programme. |
